# Revealing lactylation-mediated mechanisms and hub genes in heart failure pathogenesis

**DOI:** 10.3389/fcvm.2025.1622958

**Published:** 2025-08-12

**Authors:** Hongguang Xie, Yiqiang Wang, Xing Zhu, Lili Zhang, Heli Niu, Hongguang Jin

**Affiliations:** ^1^ School of Traditional Chinese Medicine, Baicheng Medical College, Baicheng, Jilin, China; ^2^ Department of Cardiology, The Affiliated Hospital of Changchun University of Chinese Medicine, Changchun, Jilin, China

**Keywords:** lactylation, heart failure, macrophage polarization, JAK/STAT, bioinformatics analysis

## Abstract

**Purpose:**

This study explores lactylation's pivotal role in the disease progression of heart failure (HF).

**Methods:**

The GSE57345 dataset, encompassing 177 HF samples and 136 normal controls (CTL), was sourced from Gene Expression Omnibus (GEO). Differentially expressed genes between HF and CTL groups underwent enrichment analysis using Gene Ontology (GO) and the Kyoto Encyclopedia of Genes and Genomes (KEGG) pathways. Weighted correlation network analysis (WGCNA) and unsupervised clustering were employed to identify HF-associated gene modules and subtypes, and these were intersected with lactate-related genes (LRGs), curated from the Molecular Signatures Database and GeneCards, to pinpoint hub genes implicated in lactylation-mediated HF (Lcy-HF). The least absolute shrinkage and selection operator (LASSO), XGBoost, Boruta algorithm, and protein–protein interaction (PPI) networks were utilized to identify these hub genes. The diagnostic potential and biological significance of these hub genes in HF progression were assessed using receiver operating characteristic (ROC) curves, gene set enrichment analysis (GSEA), and immune infiltration analysis.

**Results:**

In the comparison between HF and CTL samples, 91 upregulated and 88 downregulated genes were identified, primarily enriched in inflammatory responses and pathways. By intersecting 387 LRGs curated from databases, we pinpointed six hub genes implicated in Lcy-HF: GATA2, HBB, JAK2, STAT2, STAT4, and WARS2. Immune infiltration analysis further revealed that these Lcy-HF hub genes are associated with macrophage polarization.

**Conclusions:**

Lactylation plays a crucial role in the pathogenesis of HF, with genes such as GATA2, HBB, JAK2, STAT2, STAT4, and WARS2 emerging as potential lactylation biomarkers for HF identification. The lactylation-macrophage polarization–inflammation axis stands out as a pivotal mechanism driving HF progression.

## 1 Introduction

Heart failure (HF) stands as a leading cause of cardiovascular mortality, affecting over 64 million people globally, with a 5-year mortality rate exceeding 75% (1). Primarily driven by aberrant cardiac structural function and underpinned by various underlying heart conditions, HF incidence is notably higher among individuals aged 65 and above (2). The pathophysiology of HF encompasses neural activation, inflammation, oxidative stress, and aberrant energy metabolism (3). Myocardial metabolic reprogramming has been established as a central metabolic mechanism in HF, marked by a shift from aerobic fatty acid oxidation to anaerobic glycolysis. Lactate, the final product of glycolysis, has been shown to modulate inflammatory factor production in HF (4, 5). However, current research mainly centers on lactate levels, gene expression, and protein function, leaving the mechanisms of posttranslational modifications (PTMs), particularly lactylation, largely unexplored.

Lactylation, a novel posttranslational modification of histones, primarily governs gene expression through the covalent attachment of lactate to histone lysine residues (Kla) (6). Recent research has demonstrated that genes associated with lactylation can serve as biomarkers for nasopharyngeal carcinoma, rheumatoid arthritis, and acute myocardial infarction (7, 8), indicating that lactylation is not only implicated in metabolic regulation but also plays a role in tumor progression, inflammatory responses, and cardiovascular diseases. Consequently, we hypothesize that an abnormal increase in lactate levels may affect the progression of HF through lactylation, which affects inflammatory responses and regulates apoptosis. Recently, Li et al. (9) identified BRD4 as a key biomarker for HF diagnosis, but the regulatory gene network and precise mechanisms underlying lactylation's role in heart failure are still unclear.

Bioinformatics, an interdisciplinary field merging biology, computer science, and statistics, offers a powerful approach to dissecting the relationship between lactylation and HF by integrating multi-omics datasets. In this study, bioinformatics analysis combined with transcriptome was employed to construct a regulatory gene network elucidating lactylation's role in HF, and the gene regulation and molecular mechanism of lactylation in the course of HF were systematically discussed through functional enrichment analysis, protein–protein interaction (PPI) network construction, and immune microenvironment analysis. The aim is to identify potential biomarkers and therapeutic targets for HF, thereby laying a scientific foundation for precise diagnostic and treatment strategies centered around lactylation.

## 2 Materials and methods

### 2.1 Data source

The RNA-seq dataset GSE57345, comprising 177 HF samples and 136 CTL samples, was retrieved from the National Center for Biotechnology Information's Gene Expression Omnibus (GEO) database (https://www.ncbi.nlm.nih.gov/geo/). The HF samples included 144 males aged 18–75 years with a mean age of 56 years and 33 females aged 31–69 years with a mean age of 55 years. The CTL samples consisted of 73 males aged 1–79 years with a mean age of 49 years and 63 females aged 8–80 years with a mean age of 51 years. The detailed background information of the samples is shown in Supplementary Table 1. By leveraging the Molecular Signatures Database (http://www.gsea-msigdb.org/gsea/index.jsp) and the GeneCards database (https://www.genecards.org/), we identified 387 LRGs (Supplementary Table 2).

### 2.2 Differential gene analysis and functional enrichment analysis

Differential expression analysis was conducted between HF and CTL samples using the limma package (version 4.4.2) in the R language (10), and genes with |log2FC| > 0.7 and *P* < 0.05 were deemed differentially expressed genes (DEGs). These DEGs were then subjected to Gene Ontology (GO) enrichment analysis, covering biological processes, molecular functions, and cellular components, as well as KEGG pathway analysis, utilizing the “clusterProfiler” package in R software. Subsequently, the intersection between the DEGs and lactate-related genes (LRG) was calculated.

### 2.3 Analysis of immune infiltration characteristics

The CIBERSORT software (version 1.03) (11) was employed to quantify the relative abundance of 22 immune cell types in the sample. For parameter settings, the gene expression matrix was converted to transcripts per million (TPM) format, deconvolution analysis of human samples was performed using the LM22 signature matrix (22 immune cell types), 1,000 permutations were used to calculate *P*-values, and quantile normalization was enabled (QN = TRUE). Based on the gene expression matrix, the transposed convolution algorithm was utilized to delineate the composition of immune-infiltrating cells using the preset 547 barcode genes. The cumulative estimated proportions of all immune cell types within each sample summed to unity. The Wilcoxon rank-sum test was applied to assess variations in immune cell infiltration across different groups. A *P*-value below 0.05 was deemed indicative of statistically significant differences.

### 2.4 Weighted gene co-expression network analysis

Module-related genes were identified using the R package “WGCNA” (version 4.4.2) (12), and the median absolute deviation (MAD) was computed for each gene independently. In addition, the top 50% of genes with the lowest MAD values were excluded to focus on the most variable genes. Subsequently, correlation coefficients among the remaining genes were calculated, and the correlation matrix was converted into a neighborhood matrix to construct a gene co-expression network. Leveraging topological overlap, genes are clustered into distinct modules based on nearest neighbor measurements, grouping genes with inherent commonalities and similarities into their grouping genes with inherent commonalities and similarities into their respective functional modules. The module genes related to HF were obtained, and the intersection between these HF module-related genes and LRG was taken.

### 2.5 Machine-learning method and consensus clustering analysis

To pinpoint hub genes within the lactylation-mediated HF (Lcy-HF)-associated gene set, we employed three robust methodologies: least absolute shrinkage and selection operator (LASSO), Extreme Gradient Boosting (XGBoost), and the Boruta algorithm. Subsequently, unsupervised cluster analysis of HF samples was performed using the R package “ConsensusClusterPlus” (version 4.4.2), leveraging the “means” clustering method according to Lcy-HF hub genes. The sample consistency cutoff value was set at 0.8, and the feature consistency cutoff value was established at 1.

### 2.6 PPI network analysis and gene set enrichment analysis

Fifteen Lcy-HF hub genes were input into the STRING database (https://string-db.org/) to analyze protein–protein interactions (PPIs), and the PPI network was constructed using Cytoscape software (version 3.8.1). Genes with a comprehensive interaction score exceeding 0.4 were designated as hub regulatory genes. These hub genes were further analyzed through gene set enrichment analysis (GSEA), with significantly enriched pathways identified using an adjusted *P* threshold of <0.05. Differential expression analysis was conducted using the “limma” (version 4.4.2) in R, while enrichment analysis of differential genes was performed with the “clusterProfiler” (version 4.4.2) (13). Visualization of the enrichment analysis results was achieved using the “ggstatsplot” (version 4.4.2).

### 2.7 ROC curve

To assess the predictive capacity of hub genes for HF disease progression, the ROC curve was plotted using the R package “pROC” (version 4.4.2) (14), with the expression value of hub genes as the variable and the disease situation (pCR or non-pCR) as the predictor. The AUC function in the package was used to calculate the area under the curve (AUC) value.

### 2.8 The risk scoring model by LASSO

A LASSO model was developed using the “glmnet” (version 4.4.2) package in the R language. Variables associated with Lcy-HF were selected from the intersection genes, and the coefficients for each variable were computed. The selected variables and their respective coefficients were then utilized to calculate a risk score for each sample using the following formula:

−0.36∗GATA2+0.07∗HBB+0.26∗JAK2+0.36∗STAT2+0.06∗STAT4+0.38∗WARS2.

### 2.9 Statistical analysis

All bioinformatics analyses were conducted using the R language (version 4.4.2). Statistical significance was set at *P* < 0.05. For the animal experimental data, results are presented as the mean ± SD.

## 3 Results

### 3.1 Expression of DEGs and pathogenesis investigation of HF

**Figure 1:**
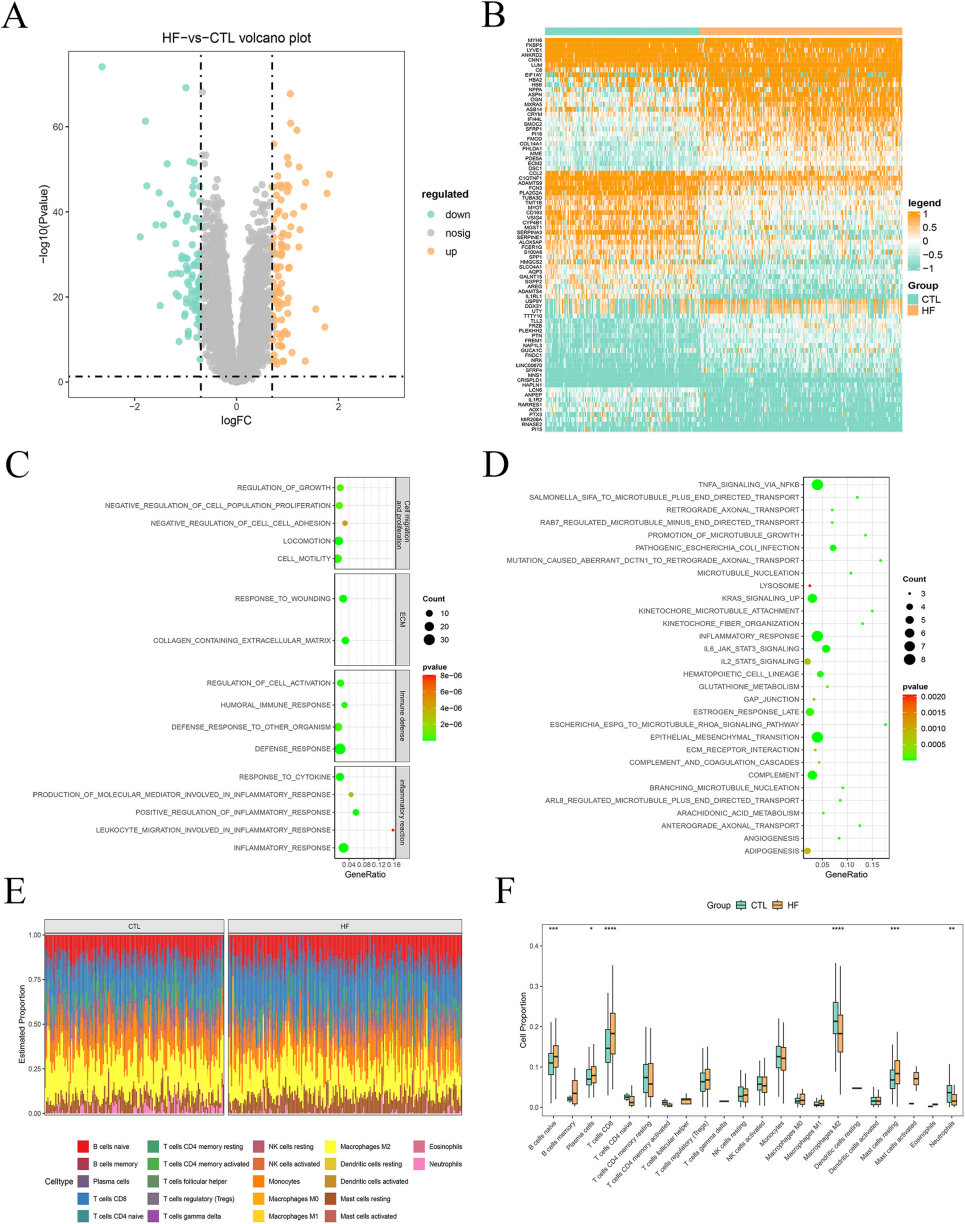
Expression of DEGs and pathogenesis investigation of HF. **(A)** Volcano plot of DEGs. **(B)** Heatmap of top 100 DEGs sorted by absolute difference multiples (R package limma, |log2FC| > 0.7, *P* < 0.05). **(C)** Bubble plot of GO functional enrichment analysis. **(D)** Bubble plot of KEGG signaling pathways enrichment analysis. **(E)** CIBERSORT-based analysis of 22 immune cell-type distributions in HF and CTL (Wilcoxon rank-sum test). **(F)** Map of immune cell distribution between CTL and HF samples.

Differential expression analysis was conducted on transcriptomic data to compare the HF and CTL groups. Applying the screening criteria of |log2FC| > 0.7 and *P* < 0.05, we identified 91 upregulated and 88 downregulated genes in HF (Figure 1A, Supplementary Table 3). The top 100 DEGs were visualized in a heatmap (Figure 1B). Functional enrichment analysis of these DEGs highlighted their regulatory roles in HF pathogenesis. GO analysis revealed that DEGs are predominantly involved in cell migration and proliferation, extracellular matrix (ECM) dynamics, immune defense, and inflammatory pathways (Figure 1C). KEGG analysis indicated that DEGs are primarily enriched in inflammatory response pathways, with notable activation of tumor necrosis factor (TNF)-α and IL6-JAK-STAT3 (Figure 1D). Furthermore, analysis of the immune microenvironment revealed significant differences in the proportions of naive B cells (*P* = 0.00024), plasma cells (*P* *=* 0.04798), CD8+ T cells (*P* *=* 1.2e-05), M2 macrophages (*P* = 3.3e-07), resting mast (*P* = 0.00019) cells, and neutrophils (*P* = 0.00362) between the two groups (Figures 1E,F). These findings suggest an enhanced inflammatory response and increased apoptosis in the heart, consistent with previous observations.

### 3.2 Identification of HF-related genes

**Figure 2:**
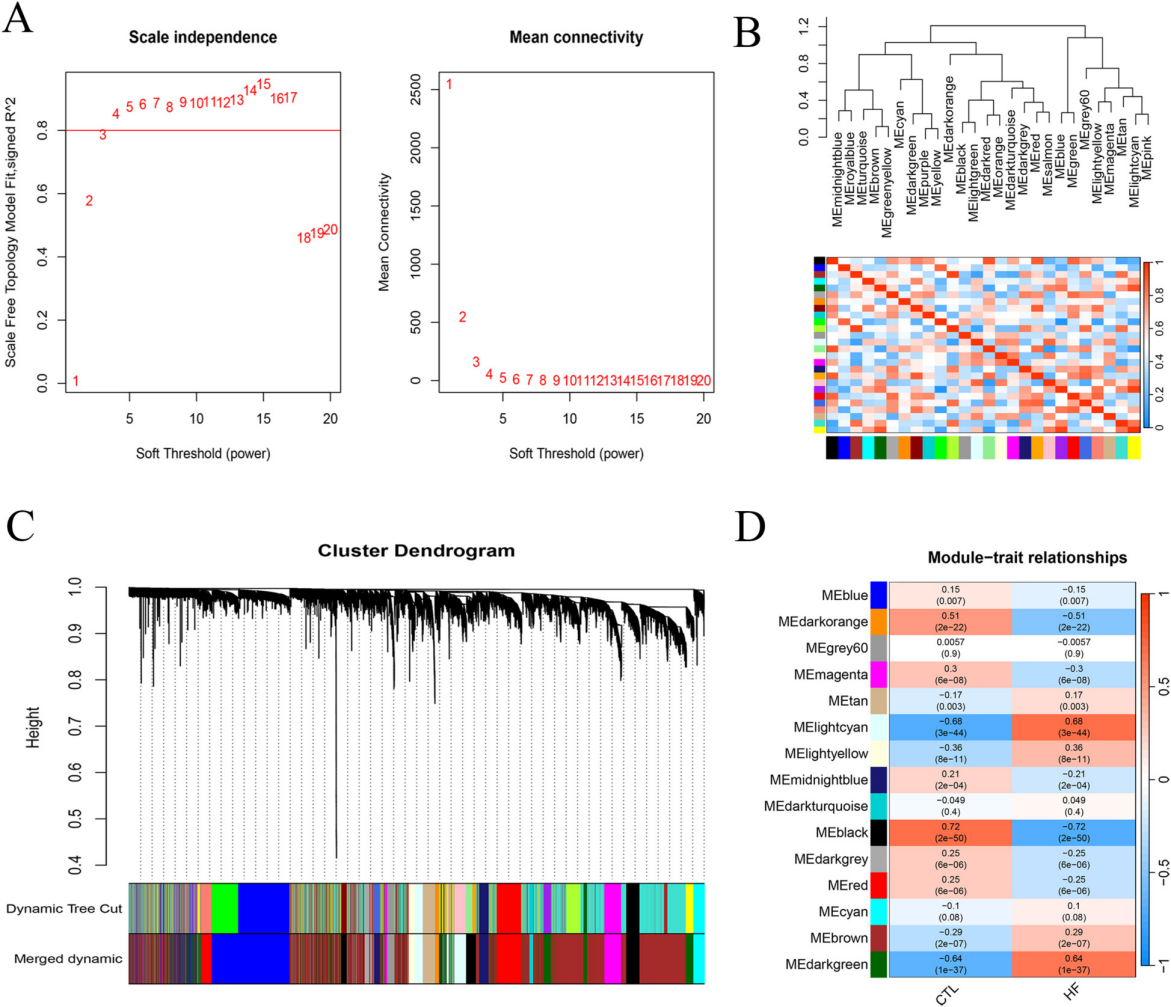
Identification of HF-related genes. **(A)** WGCNA analysis plot of soft threshold *β* = 0.5 (horizontal axis, soft threshold; vertical axis, scale-free fitting index; R package WGCNA). **(B)** Correlation heatmap of 27 gene modules. **(C)** Gene module clustering plot (top, gene hierarchical clustering dendrogram; bottom, gene modules, distinguished by different colors). **(D)** Heatmap depicting the association between gene modules and HF.

To further elucidate gene modules associated with HF, the gene expression matrix was used for WGCNA analysis, and the gene network was constructed when the soft threshold *β* = 5 (Figure 2A). Twenty-seven gene modules were identified, which were subsequently merged into 15 modules based on correlation coefficients exceeding 0.75 (Figures 2B,C). Next, the correlations between gene modules and HF and CTL were calculated (Figure 2D), and the Lightcyan and Darkgreen modules demonstrated significant associations with HF (*P* < 0.05). Therefore, these modules were selected for further analysis as HF-related gene modules.

### 3.3 Identification of Lcy-HF hub genes

**Figure 3:**
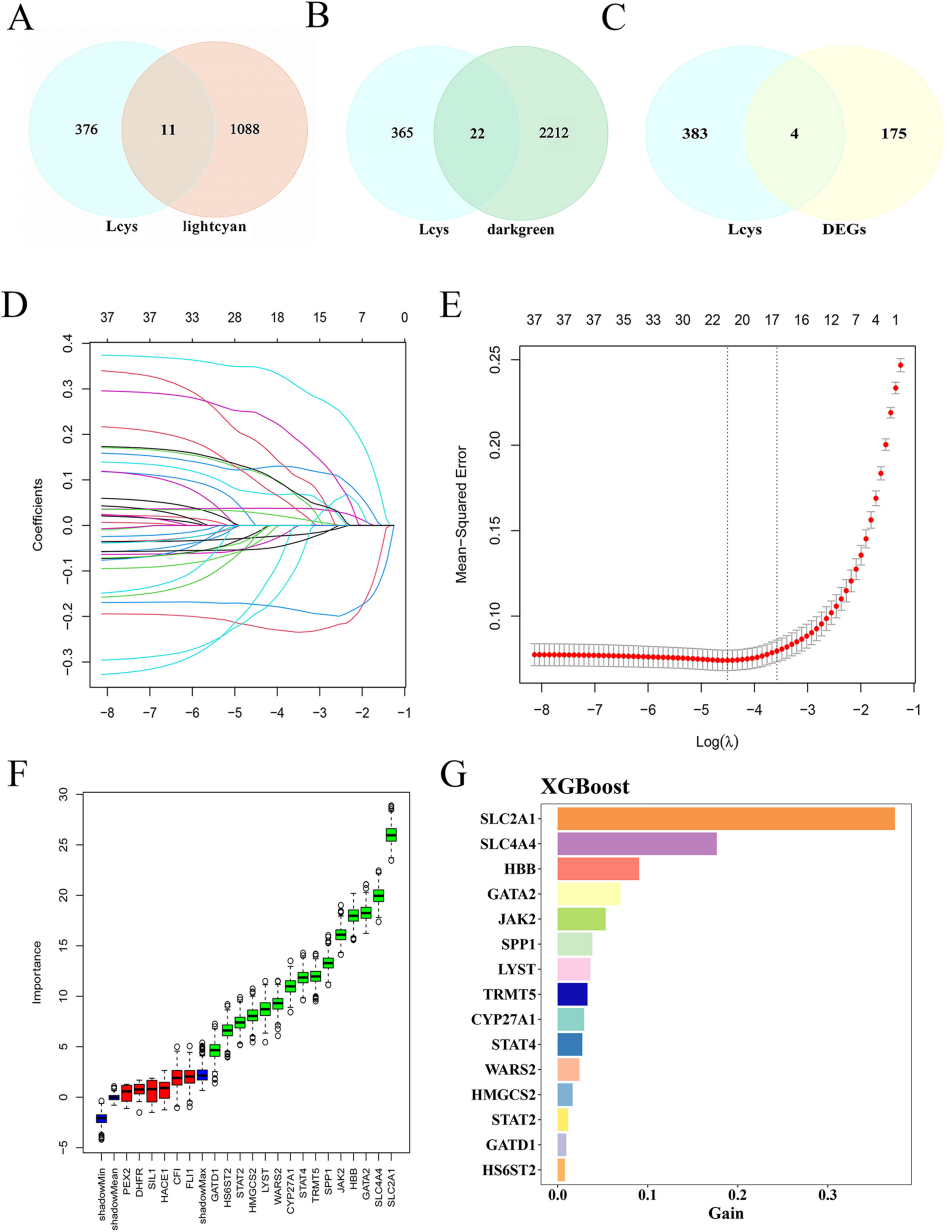
Identification of Lcy-HF hub genes. **(A)** Venn diagram illustrating the intersection between the Lightcyan module and LRG. **(B)** Venn diagram displaying the intersection between the Darkgreen module and LRG. **(C)** Venn diagram of DEGs and LRG. **(D)** Plot with the horizontal axis representing the logarithm of the regularization parameter *λ* (lambda) in LASSO regression, and the vertical axis showing the coefficient value of each feature (variable). Each line depicts the trajectory of a feature's coefficient change with respect to *λ*. **(E)** LASSO regression plot of mean square error (R package glmnet). **(F)** Plot generated by the Boruta algorithm. **(G)** XGBoost-based ranking of feature gene importance.

The Lightcyan and Darkgreen gene modules intersected with LRG, yielding 11 and 22 Lcy-HF-related genes (Figures 3A,B). Additionally, intersecting the 179 DEGs in HF with LRGs identified 4 Lcy-HF-related genes. By integrating these findings, we pinpointed 37 genes linked to Lcy-HF (Figure 3C, Supplementary Table 4). Subsequently, three established machine-learning methods were employed to further screen and identify the hub genes within the Lcy-HF network, and the results show that LASSO regression analysis identified 21 genes associated with HF (Figures 3D,E). Among these, the Boruta algorithm was utilized to select 15 hub genes (Figure 3F), which were then validated and ranked using the XGBoost (Figure 3G). In addition, among these hub genes, 10 were upregulated (CYP27A1, GATD1, HBB, JAK2, LYST, STAT2, STAT4, TRMT5, and WARS2) and 5 were downregulated (GATA2, HMGCS2, HS6ST2, SLC2A1, and SPP1) in HF (Supplementary Figure 1).

### 3.4 Categorization of Lcy-HF-related subtypes and functional enrichment analysis

**Figure 4:**
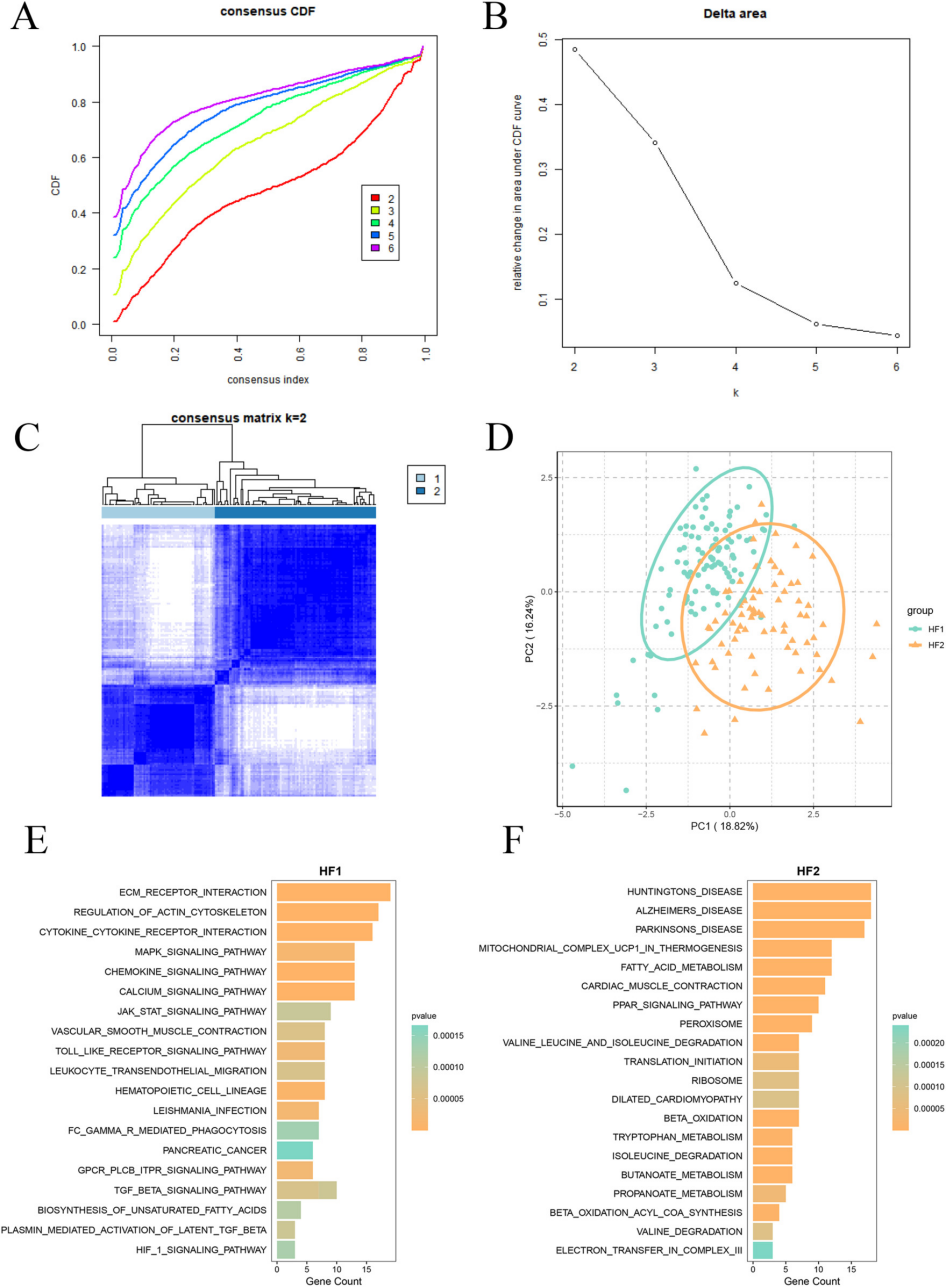
Categorization of Lcy-HF-related subtypes and functional enrichment analysis. **(A)** Consensus cumulative distribution function (CDF) plot (*k* = 2–6). **(B)** Plot depicting the relative change in the area under the CDF curve (*k* = 2–6). **(C)** Cluster analysis heatmap (*k* = 2). **(D)** PCA plot for HF1 and HF2 subtypes. **(E)** Column diagram illustrating functional enrichment analysis for HF1 subtype. **(F)** Functional enrichment analysis outcomes for the HF2 subtype.

To further delineate Lcy-HF subgroups, we performed consensus clustering on 15 identified Lcy-HF hub genes. As illustrated in Figures 4A,B, when the consensus matrix number was set to 2, the cumulative distribution function (CDF) curve exhibited minimal fluctuation across the consistency index range of 0–1.0, indicating the most stable number of subtypes, the largest area under the CDF curve, and a more pronounced clustering effect. Therefore, we selected *k* = 2 and ultimately classified the samples into two distinct subtypes (Figure 4C), which we named HF1 and HF2. Further PCA confirmed substantial differences between the HF1 subgroup (Figure 4D). Functional enrichment analysis revealed that MAPK, JAK/STAT, and TGF-β signaling pathways were significantly enriched in the HF1 subtype (Figure 4E), whereas the HF2 subtype was predominantly associated with mitochondrial energy metabolism (Figure 4F).

### 3.5 Identification of hub genes in PPI networks

**Figure 5:**
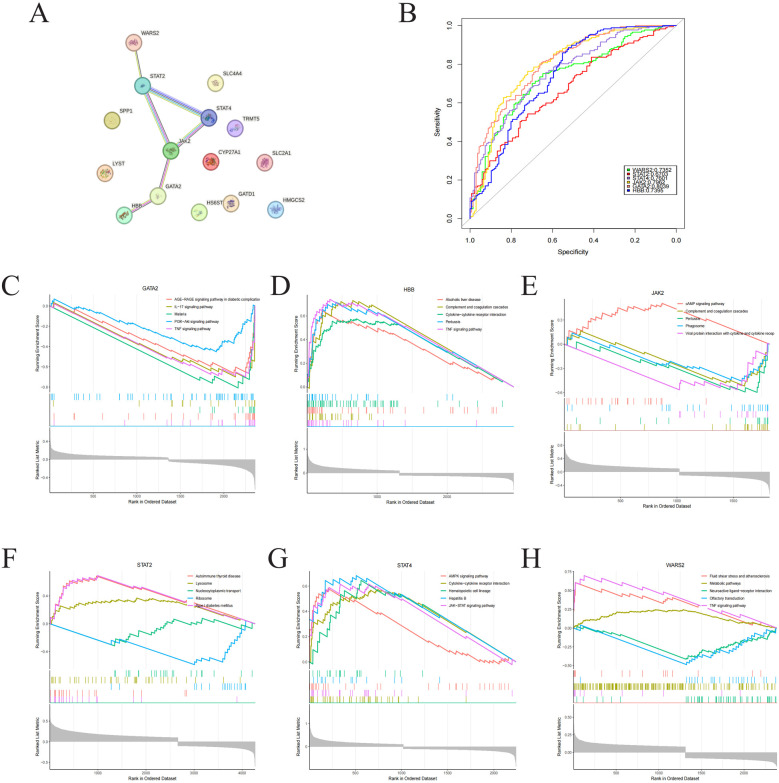
Identification of hub genes in PPI networks. **(A)** PPI network visualization of 15 Lcy-HF hub genes. **(B)** ROC curve presentation (R pROC) for six Lcy-HF hub genes: GATA2 (95% CI: 0.7555–0.8523), HBB (95% CI: 0.6824–0.7967), JAK2 (95% CI: 0.7448–0.8477), STAT2 (95% CI: 0.611–0.7297), STAT4 (95% CI: 0.7076–0.8126), WARS2 (95% CI: 0.6796–0.7908). **(C–H)** GSEA enrichment analysis (R limma) results for the six Lcy-HF hub genes (GATA2, HBB, JAK2, STAT2, STAT4, WARS2).

PPI network was constructed and evaluated the interactions among 15 identified Lcy-HF hub genes, and the analysis pinpointed six dominant genes (GATA2, HBB, JAK2, STAT2, STAT4, and WARS2) within the interaction network (Figure 5A). Furthermore, ROC curve analysis underscored the strong diagnostic potential of these six hub genes (Figure 5B). To elucidate the functional roles of these six Lcy-HF hub genes in HF, GSEA was conducted, revealing their involvement in TNF and JAK/STAT signaling pathways (Figures 5C–H).

### 3.6 Relationship between Lcy-HF and immune microenvironment

**Figure 6:**
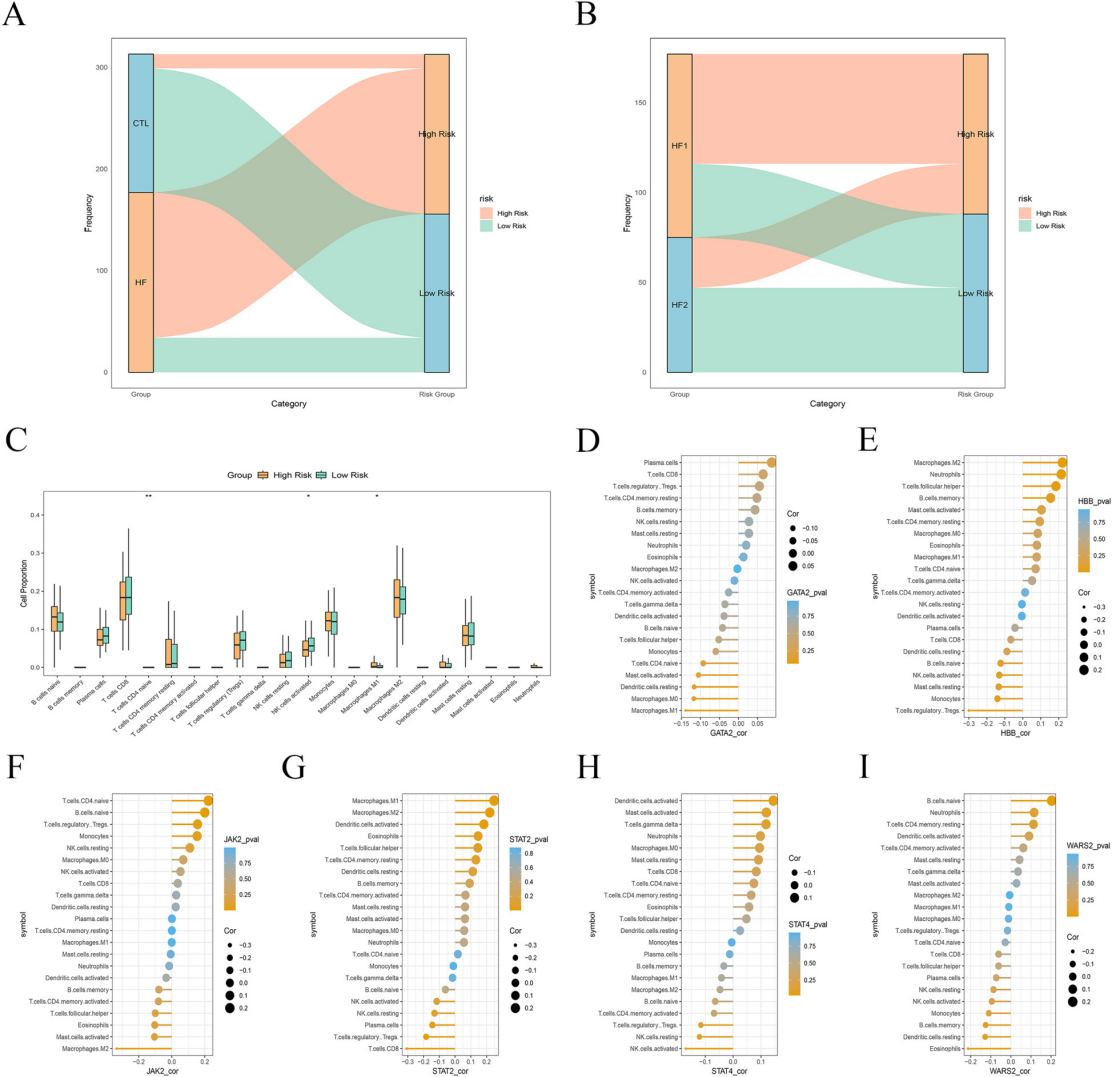
Relationship between Lcy-HF and immune microenvironment. **(A)** Risk scores impact plot comparison between CTL and HF samples. **(B)** Risk scores impact plot for HF1 and HF2 subtypes. **(C)** Distribution map of immune cells across the high- and lower-rank groups. **(D–I)** Correlation analysis results between six hub genes and immune cells (GATA2, HBB, JAK2, STAT2, STAT4, WARS2).

LASSO regression method was employed to construct a Lcy-HF risk score model based on six Lcy-HF genes, and they were stratified into high- and low-risk groups using the median expression value as the cutoff. Upon analyzing “CTL and HF samples” as well as “HF1 and HF2 subtypes,” we observed that a greater proportion of HF samples were classified into the high-risk group, whereas CTL samples predominantly fell into the low-risk group (Figure 6A). Similarly, within the HF subtypes, more HF1 samples were categorized as high-risk, while HF2 samples were more frequently classified as low-risk (Figure 6B), further confirming that HF1 is associated with poor HF outcomes. Immunoinfiltration analysis of the high- and low-risk groups revealed a higher proportion of M1 macrophages (*P* = 0.0341), CD4+ T cells (*P* = 0.0055) and NK cells (*P* = 0.0251) in the high-risk group (Figure 6C), while correlation analysis of the six Lcy-HF genes and immune cells demonstrated a positive correlation between STAT2 and M1 macrophages (Figures 6D–I). This suggests that the Lcy-HF gene STAT2 may exacerbate HF progression by promoting M1 macrophage polarization and amplifying the inflammatory response.

## 4 Discussion

HF is characterized by the heart's inability to adequately meet the body's metabolic demands, primarily manifesting as reduced oxidation of fatty acids and glucose alongside increased reliance on lactic acid oxidation and glycolysis (15). Despite advancements in treatment, such as the use of angiotensin-converting enzyme inhibitors, angiotensin receptor blockers, and β-blockers, which have significantly improved patient outcomes (3), the mortality rate among patients remains high, and therapeutic challenges persist (16). Lactylation, a PTM that links cellular metabolism with epigenetics and signaling pathways, primarily involves the transfer of the lactyl moiety from lactic acid to the ε-amino group of lysine residues. This modification exists in both histones and non-histone proteins. Histone lactylation relies on L-lactate-induced enzymatic reactions, regulating chromatin conformation to modulate gene transcription. In contrast, non-histone lactylation primarily affects protein functions by regulating enzymatic activities (17). In this study, bioinformatics combined with transcriptomic analysis systematically revealed the molecular mechanism and hub gene regulation of lactylation in the progression of HF.

GO and KEGG analyses were conducted on the 179 DEGs identified between the HF and CTL groups. These DEGs were predominantly found to be enriched in pathways associated with the inflammatory response and related signaling pathways, such as IL-6 and JAK/STAT. The downregulation of M2 macrophages was observed through immune infiltration analysis, indicating heightened myocardial inflammation in HF. The elevation of tumor necrosis factor-α (TNF-α) and IL-6 levels, along with the occurrence of inflammatory responses, has been extensively documented in HF, though the diverse causes underlying these inflammatory responses remain a subject of ongoing investigation (18). Current studies have elucidated that elevated lactate levels can modulate inflammatory factors in cardiac fibroblasts (5) and are also associated with mortality in HF patients (19). Lactic acid can be converted into lactoyl-CoA, participate in protein posttranslational modifications (lactylation), regulate macrophage polarization and inflammatory factor secretion, maintain sarcomeric structure and function, and ultimately affect the progression of HF (20, 21). Reduction in lactate production decreases H3K18 lactylation expression, inhibits myocardial cell hypertrophy, and reduces mortality in HF (22). Moreover, the lactate dehydrogenase encoding gene LDHA, which catalyzes the conversion between pyruvate and lactic acid, can induce myocardial pyroptosis by enhancing NLRP3 lactation, thereby promoting myocardial ischemia–reperfusion injury (23). Monocarboxylate transporter 4 (MCT4), responsible for exporting intracellular lactate, is abnormally upregulated on the plasma membrane of cardiomyocytes in diabetic cardiomyopathy, leading to excessive efflux of intracellular lactate. This process increases histone H4K12 lactylation in macrophages, promoting inflammatory infiltration in the microenvironment, which confirms the regulatory role of lactylation in inflammation (24).

Fifteen genes were identified as Lcy-HF genes, suggesting their potential involvement in HF progression through lactylation. Enrichment analysis of the lactylation–heart failure subsets (HF1 and HF2), which were delineated by these Lcy-HF genes, indicated that lactylation might modulate cardiomyocyte apoptosis and facilitate cardiac fibrosis by modifying kinases in the MAPK pathway and TGF-β proteins (25, 26). Concurrently, in alignment with the differential gene enrichment analysis of HF samples, an enhanced inflammatory response was observed through the JAK/STAT pathway, exacerbating the myocardial injury. The JAK/STAT signaling pathway, known for its regulatory role in HF, comprises members such as JAK2, STAT1, STAT2, STAT3, and STAT4 (27). Previous studies have demonstrated that JAK2/STAT1 can be used as a therapeutic target for HF (28), while a complete deficiency of STAT2 can lead to inflammatory diseases (29). STAT4 has been proposed as a potential biomarker for HF comorbid with depression. However, our analysis, for the first time, revealed that JAK2, STAT2, and STAT4 can be utilized as lactylation hub regulatory genes to identify HF. Moreover, GATA2, HBB, and WARS2 were also identified as diagnostic markers for HF, a finding supported by ROC curve analysis. GATA2, a transcription factor of the GATA family, has been shown to regulate miRNA to promote cardiac fibrosis (30) and aggravate HF induced by experimental transverse aortic coarctation after GATA2 knockdown (31). Consistent with our findings, GATA2 was found to be significantly downregulated in HF. Recent studies have indicated that the downregulation of GATA2 reduces the pro-inflammatory phenotype of pulmonary macrophages in chronic obstructive pulmonary disease (32), but no significant association between GATA2 and macrophages has been previously reported in HF, and the specific underlying mechanism warrants further investigation. The hemoglobin beta subunit (HBB) and angiogenesis factor (Was2) have also been identified as potential biomarkers for HF (33, 34).

Interestingly, the GSEA of six hub Lcy-HF genes revealed their enrichment in the JAK/STAT signaling pathway. Previous studies have shown that when lactylation occurs in the catalytic or regulatory domains of kinases, the negatively charged lactate moiety alters the local charge distribution, leading to conformational changes in the kinase. For example, increased lactylation of PKM2 at K62 directly drives PKM2 into a more active tetrameric form and enhances its pyruvate kinase activity. Lactate suppresses the Warburg effect by activating PKM2, thereby promoting the transition of pro-inflammatory macrophages to a reparative phenotype (35). Lactylation of Vps34 enhances its binding to Beclin1, Atg14l, and UVRAG, thereby increasing Vps34 lipid kinase activity and regulating cellular autophagy. During intense exercise, Vps34 lactylation in skeletal muscle maintains muscle cell homeostasis (36). Additionally, lactylation indirectly affects kinase activity. Wang et al. (37) found that reducing H3K18 lactylation at the IRS1 promoter decreases IRS1 expression, weakens the activities of PI3 K/AKT/mTOR and MAPK/ERK pathways, and suppresses the growth and metastasis of hepatocellular carcinoma cells. Therefore, we hypothesize that lactylation may directly or indirectly regulate the JAK/STAT signaling pathway to exert effects on HF, and the specific mechanism requires further in-depth investigation. Furthermore, correlation analysis demonstrated a positive correlation between STAT2 and M1 macrophages. This is consistent with previous findings that STAT2 regulates macrophage polarization phenotypes during influenza–bacterial coinfection and in multiple myeloma (38, 39). However, no studies have explored the association between STAT2 and macrophage polarization in heart failure. Future research should validate lactylation-induced macrophage polarization through STAT2 knockdown experiments and further verification.

Finally, immunoinfiltration analysis was conducted among the risk groups established using a risk score model for lactylation in HF, which was constructed based on six Lcy-HF genes. The observed increase in M1 macrophages further suggested that lactylation might promote the M1 polarization of macrophages, thereby exerting a pro-inflammatory effect in HF. The JAK/STAT signaling pathway is also recognized as a key player in macrophage polarization (40). It is therefore proposed that lactylation may regulate M1 macrophage polarization by modulating the JAK/STAT2 signaling pathway, thereby promoting an inflammatory response that contributes to HF progression. Previous clinical studies review suggests that the immune response in HF is a secondary phenomenon in response to myocyte injury (41). The mechanism underlying HF is complex and multifaceted. In HF, lactylation may not be a cause but rather a consequence of macrophage polarization, occurring concurrently with metabolic reprogramming induced by inflammatory stress (42). Although the regulatory role of lactylation in macrophage polarization requires further investigation, the relationship between lactylation and inflammation is certainly.

Although this study employed bioinformatics to analyze the mechanism of lactylation in HF, certain limitations persist. Firstly, the findings derived from public databases necessitate experimental validation, and we did not perform external validation to confirm the AUC performance of the diagnostic model, which limits the generalizability of our findings to other populations or clinical settings. Secondly, the specific sites of lactylation remain insufficiently explored, and a deeper understanding of the regulatory mechanisms of histone and non-histone lactylation in HF is warranted. Finally, the effects of lactic acid metabolism in intestinal flora on homeostasis and the progression of HF were not considered in the study. In future research, emphasis can be placed on investigating how group lactylation influences the regulatory mechanisms of macrophage polarization in the pathogenesis of HF, potentially offering a novel therapeutic target for this condition.

## 5 Conclusions

In summary, lactylation may contribute to the progression of HF by regulating macrophage polarization and promoting inflammatory response. GATA2, HBB, JAK2, STAT2, STAT4, and WARS2 have been identified as potential novel biomarkers to identify HF.

## Data Availability

Publicly available datasets were analyzed in this study. This data can be found here: https://www.ncbi.nlm.nih.gov/geo/query/acc.cgi?acc=GSE57345.

## Supplementary material

Supplementary Figure 1 Expression of 15 Lcy-HF hub genes in HF and control samples.
