# Supplementary figures and images for: Revealing lactylation-mediated mechanisms and hub genes in heart failure pathogenesis
Source: Front Cardiovasc Med. 2025 Aug 12;12:1622958. doi: 10.3389/fcvm.2025.1622958 (PMC12378386; doi:10.3389/fcvm.2025.1622958)

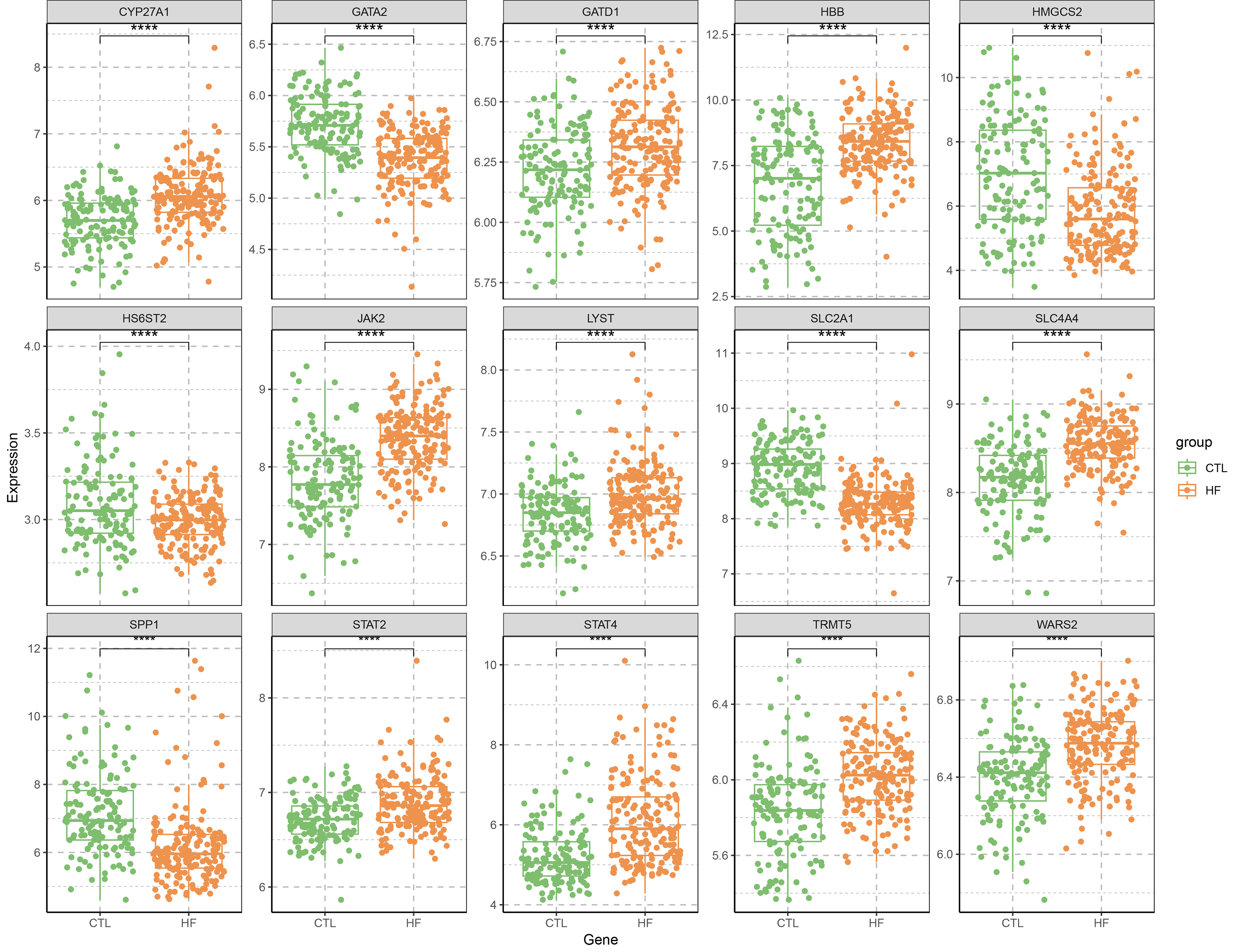

Supplement: Supplementary Figure 1 — Expression of 15 Lcy-HF hub genes in HF and control samples. [file Image1.tif]
